# Race-specific association of an *IRGM* risk allele with cytokine expression in human subjects

**DOI:** 10.1038/s41598-023-40313-3

**Published:** 2023-08-09

**Authors:** Teminioluwa Ajayi, Prashant Rai, Min Shi, Kristin A. Gabor, Peer W. F. Karmaus, Julie M. Meacham, Kevin Katen, Jennifer H. Madenspacher, Shepherd H. Schurman, Michael B. Fessler

**Affiliations:** 1 Immunity, Inflammation and Disease Laboratory, National Institute of Environmental Health Sciences, 111 T.W. Alexander Drive, MD D2-01, P.O. Box 12233, Research Triangle Park, NC 27709 USA; 2 Biostatistics & Computational Biology Branch, National Institute of Environmental Health Sciences, Research Triangle Park, NC 27709 USA; 3 Signal Transduction Laboratory, National Institute of Environmental Health Sciences, Research Triangle Park, NC 27709 USA; 4 Clinical Research Branch, National Institute of Environmental Health Sciences, Research Triangle Park, NC 27709 USA; 5 Present Address: Clinical Research Unit, National Institute on Aging, Baltimore, MD 21225 USA

**Keywords:** Translational immunology, Genetic association study, Preclinical research

## Abstract

Immunity-related GTPase family M (*IRGM*), located on human chromosome 5q33.1, encodes a protein that promotes autophagy and suppresses the innate immune response. The minor allele of rs13361189 (−4299T>C), a single nucleotide polymorphism in the *IRGM* promoter, has been associated with several diseases, including Crohn’s disease and tuberculosis. Although patterns of linkage disequilibrium and minor allele frequency for this polymorphism differ dramatically between subjects of European and African descent, studies of rs13361189 have predominantly been conducted in Europeans and the mechanism of association is poorly understood. We recruited a cohort of 68 individuals (30 White, 34 African American, 4 other race) with varying rs13361189 genotypes and assessed a panel of immune response measures including whole blood cytokine induction following ex vivo stimulation with Toll-like Receptor ligands. Minor allele carriers were found to have increased serum immunoglobulin M, C-reactive protein, and circulating CD8^+^ T cells. No differences in whole blood cytokines were observed between minor allele carriers and non-carriers in the overall study population; however, minor allele status was associated with increased induction of a subset of cytokines among African American subjects, and decreased induction among White subjects. These findings underline the importance of broad racial inclusion in genetic studies of immunity.

## Introduction

Genes have been estimated to account for ~ 20–40% of the variation in immunologic function across humans, contributing to temporally stable immunologic phenotypes^1,2^. Identifying genetic polymorphisms that modify disease risk through altering immune cell function is one of the tenets of modern personalized medicine. Multiple genes orchestrate autophagy, an evolutionarily conserved process by which cells clear invasive pathogens and damaged organelles. Autophagy has been shown to suppress the pro-inflammatory functions of immune cells through multiple mechanisms, and also to serve as an effector arm of cell-autonomous antimicrobial host defense^3,4^. A growing number of polymorphisms in autophagy-related genes have recently been associated with inflammatory and infectious diseases^5–7^.

Immunity-related GTPase family M (*IRGM*), located on human chromosome 5q33.1, encodes a protein that plays a central role in autophagy by coupling core autophagy proteins to innate immunity receptors^8^. *IRGM* is required for autophagy induction by bacterial lipopolysaccharide (LPS) and for autophagic killing of intracellular bacteria^9–12^. Silencing *IRGM* by RNA interference augments LPS induction of TNFα and IL-1β through increasing activation of nuclear factor-κB and p38 and stabilizing NLRP3^9,13^. *IRGM* silencing also reduces autophagic degradation of TLR3, RIG-I, and CGAS, thereby augmenting immune responses to ligands for these nucleic acid receptors^14^.

Several reports have shown that rs13361189 (−4299T>C) and rs10065172 (+313C>T), two single nucleotide polymorphisms (SNPs) that are in high linkage disequilibrium (LD) with a presumed causal 20 kb copy number variation (CNV) deletion upstream of *IRGM*, associate with increased risk for Crohn’s disease (CD)^12,15–19^. rs13361189 has also been associated with leprosy^20^ and autoimmune thyroid disease^21^, and rs10065172 with tuberculosis^22^ and sepsis mortality^23^, collectively suggesting that IRGM regulates a wide spectrum of human disease. A major limitation of the studies performed to date on *IRGM* and CD risk, however, is that nearly all have been conducted in Europeans^17,24,25^. Compared to Europeans, Africans have much higher *IRGM* expression and *IRGM* rs13361189 risk (C) allele frequency (~ 0.50 vs. 0.10 [useast.ensembl.org]), and much lower LD of rs13361189 to the upstream CNV deletion^16^.

We and others have reported that *IRGM* expression is reduced in immune cells of subjects with the rs13361189 risk allele^13,16^, suggesting that changes in *IRGM* expression may mediate the association of the risk allele with disease by impacting immune cell function. To date, few reports have investigated whether rs13361189 associates with altered cytokine induction, with divergent results and few stimuli tested^20,26,27^. Higher IFN-γ and IL-4, but unchanged IL-6 and IL-1β were seen in peripheral blood mononuclear cells from Asian risk allele carriers after *M. leprae* infection^20^, whereas minimal differences in cytokines were seen after *C. albicans*^27^*,* and none after *M. tuberculosis*^26^ in two papers that did not report race.

To better define possible race-specific effects of *IRGM* polymorphism on immune cell function, we recruited 30 White and 34 African American (AA) healthy subjects in the NIEHS Personalized Environment and Genes Study (PEGS), a North Carolina-based study of > 18,000 subjects with archived DNA^13,28^. Subjects were immunophenotyped, including cytokine profiling of whole blood. We report that rs13361189 risk (C) allele carriers have a race-specific alteration in cytokines. Specifically, risk allele status is associated with increased induction of a small subset of cytokines in AA subjects, but reduced induction among White subjects. We propose that our findings have implications for IRGM biology and that they underline the importance of broad racial inclusion in studies of genetic determinants of disease.

## Results

### Study cohort of human subjects with varying *IRGM* genotypes

A cohort of subjects with varying genotypes at rs13361189 was prospectively recruited to investigate the effect of *IRGM* polymorphism on immune phenotypes. Exclusion criteria included age < 18 years, active smoking, use of anti-inflammatory or immunosuppressive medications, confirmed/suspected immunodeficiency, and recent gastrointestinal or respiratory illness. As shown in Table 1, a predominantly female cohort of 68 individuals (n = 35 [T/T]; n = 20 [T/C]; n = 13 [C/C]) was recruited. Age and sex were comparable across genotypes. Whereas subjects with a rs13361189 homozygous major allele genotype T/T were predominantly (60%) White, consistent with the racial composition of the PEGS study population (https://www.niehs.nih.gov/research/clinical/studies/pegs/index.cfm), the two minor allele genotypes (T/C, C/C) were predominantly AA, likely reflecting the higher minor allele frequency at rs13361189 in AAs (http://useast.ensembl.org).

**Table 1:** Study population.

rs13361889 genotype	T/T	T/C	C/C	p-value*
Population (n)	35	20	13	
Sex				0.71
Male (%)	34.3	45.0	30.8	
Female (%)	65.7	55.0	69.2	
Age, years (s.d.)	52.7 (12.8)	49.4 (10.9)	49.3 (9.8)	0.63
Race				0.02
White (%)	60.0	30.0	23.1	
Black (%)	37.1	65.0	61.5	
Asian (%)	2.9	5.0	0	
Multiple race (%)	0	0	15.4	

*Fisher’s exact test was used for sex and race; ANOVA was used for age.

### Static immune phenotypes across *IRGM* genotypes

Static immune metrics were first assessed, including complete blood cell count with differential, serum C-reactive protein (CRP) concentration, serum immunoglobulin (Ig) levels, and peripheral blood mononuclear cell profile by flow cytometry (Figs. S1, S2). Here and in all analyses that follow, we selected a dominant genetic model, comparing the T/T genotype to minor allele carriers (i.e., T/C and C/C subjects) by robust linear regression. This model was chosen both because of our prior finding of reduced whole blood *IRGM* expression in minor allele carriers^13^ and because of the limited N, in particular, for C/C subjects. Of note, as shown in Table 2, minor allele carriers had higher serum IgM than T/T subjects. Minor allele carriers also had numerical elevations of CRP (p = 0.06), an inflammatory biomarker, and CD8^+^ T cell count (p = 0.06). Upon race stratification, no significant differences between minor allele carriers and non-carriers were noted among AA subjects. Among White subjects, minor allele carriers exhibited increases in IgM (p = 0.02), IgE (p = 0.05), and CRP (p = 0.06), and a decrease in percentage of circulating plasmablasts (p = 0.005) (data not shown).

**Table 2:** Clinical laboratory and peripheral blood lymphocyte cytometry results.

Variable	Estimate	Std. error	t value	p-value
White blood cell count	0.238	0.405	0.587	0.562
Hemoglobin	− 0.097	0.283	− 0.342	0.732
Platelet count	12.277	13.443	0.913	0.367
Neutrophil count	114.826	333.831	0.344	0.734
Lymphocyte count	115.130	139.605	0.825	0.419
Monocyte count	25.848	31.672	0.816	0.424
Eosinophil count	− 13.376	18.983	− 0.705	0.481
Basophil count	1.695	3.307	0.512	0.611
CRP	1.003	0.510	1.968	0.063
IgA	2.268	26.244	0.086	0.931
IgE	8.258	10.727	0.770	0.455
IgG	27.086	63.905	0.424	0.676
IgM	22.459	10.172	2.208	0.031
CD27− SM B cells^a^	0.001	0.001	1.083	0.288
IgD− B cells^a^	0.006	0.005	1.164	0.254
Plasmablasts^a^	0.000	0.000	− 0.947	0.350
Naïve B cells^a^	0.020	0.019	1.047	0.298
Transitional B cells^a^	0.000	0.001	0.297	0.767
UM B cells^a^	0.001	0.003	0.428	0.677
CD3+ T cells^a^	0.182	0.102	1.792	0.083
CD14+ monocytes^a^	0.014	0.031	0.436	0.665
CD4+ T cells^a^	0.079	0.079	0.995	0.333
CD8+ T cells^a^	0.070	0.036	1.923	0.062
CD19+ B cells^a^	0.031	0.021	1.451	0.154
CD56+ NK cells^a^	0.032	0.027	1.184	0.243
Basophils^a^	− 0.002	0.005	− 0.383	0.702
Eosinophils^a^	− 0.016	0.017	− 0.933	0.349
Neutrophils^a^	0.034	0.356	0.096	0.924

For depicted variables, differences were tested between rs13361189 C allele noncarriers and carriers using robust linear regression.

^a^These results were derived from flow cytometry.

**Figure 1:**
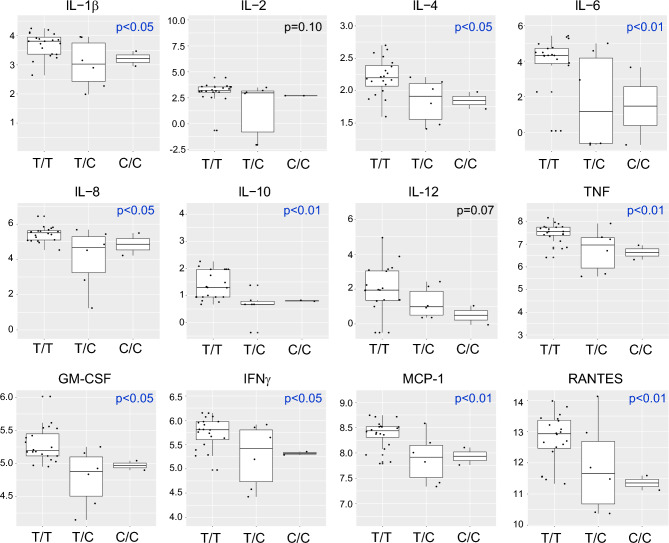
Cytokine levels in unstimulated whole blood of White subjects of different rs13361189 genotypes. Whole blood from 30 White subjects of the rs13361189 genotypes shown (n = 20 [T/T]; n = 6 [T/C]; n = 2 [C/C]) was incubated ex vivo for 3 h, after which cytokine concentrations were quantified by multiplex assay. Box and whisker plots depict median and interquartile ranges, and y-axis units depict log-transformed pg/mL values. P values were determined by robust linear regression using a dominant genetic model (T/T vs. T/C and C/C).

In order to assay steady state cytokine production by circulating immune cells, whole blood was incubated ex vivo at 37 °C for 3 h, after which the cells were centrifuged and 13 cytokines were quantified in the serum fraction by multiplex assay. As shown in Table S1, no significant differences were observed between T/T and minor allele subjects in the overall cohort. Hypothesizing that genotype-dependent effects on cytokine production might be race-specific, we analyzed steady state cytokine levels after race stratification. Of interest, we found that, among White subjects, nearly all of the cytokines assayed exhibited significant reductions in minor allele carriers (Table S2, Fig. 1). Although robust analysis of White C/C subjects was prevented by their very small number (n = 2), some cytokines exhibited potential allele dose-dependent effects, with lowest levels in C/C subjects (TNF, IL-12, RANTES, IL-4).

**Figure 2:**
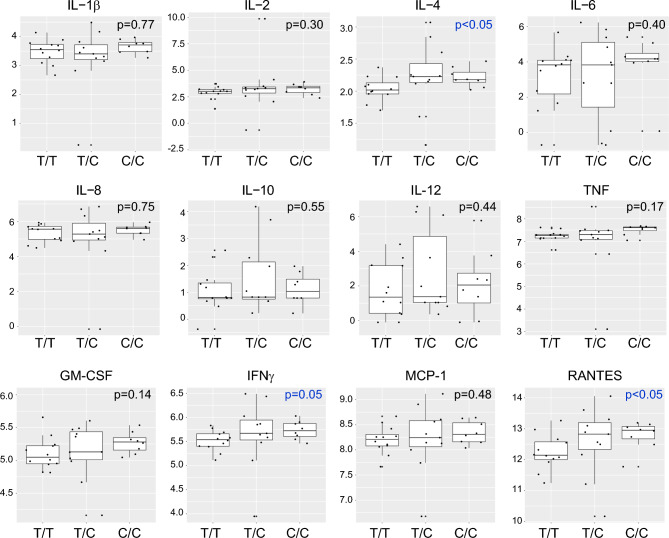
Cytokine levels in unstimulated whole blood of African American subjects of different rs13361189 genotypes. Whole blood from 34 African American subjects of the rs13361189 genotypes shown (n = 12 [T/T]; n = 11 [T/C]; n = 8 [C/C]) was incubated ex vivo for 3 h, after which cytokine concentrations were quantified by multiplex assay. Box and whisker plots depict median and interquartile ranges, and y-axis units depict log-transformed pg/mL values. P values were determined by robust linear regression using a dominant genetic model (T/T vs. T/C and C/C).

Conversely, among AA subjects, a subset of cytokines was increased among minor allele carriers, namely, IL-4, IFNγ, and RANTES (Table S3, Fig. 2). Race interaction p-values were highly significant for nearly all of the cytokines (Table 3), suggesting that the minor allele-cytokine relationship is modified by race. Taken together, these findings suggest that there is a generally opposite relationship of the rs13361189 minor allele to steady state cytokine levels in AA and White subjects, with increases in some cytokines in AA minor allele carriers and decreased cytokines in White minor allele carriers.

**Table 3:** Race interactions in control cytokine levels.

Analyte	Estimate	Std. error	t value	p-value
GM-CSF	− 0.545	0.156	− 3.487	0.001
IL-1b	− 0.658	0.260	− 2.531	0.017
IL-12	− 1.374	0.961	− 1.430	0.155
IL-2	− 0.672	0.315	− 2.135	0.042
IFN-γ	− 0.629	0.190	− 3.301	0.002
IL-10	− 0.884	0.331	− 2.669	0.010
IL-8	− 0.715	0.327	− 2.187	0.041
IP-10	− 0.409	0.231	− 1.775	0.086
MCP-1	− 0.603	0.189	− 3.194	0.003
IL-4	− 0.560	0.170	− 3.290	0.002
IL-6	− 3.114	1.128	− 2.760	0.008
TNF-α	− 0.923	0.208	− 4.445	< 0.001
RANTES	− 2.045	0.439	− 4.662	< 0.001

Cytokine levels in White rs13361189 C allele carriers minus non-C allele carriers vs. cytokine levels in African American C allele carriers minus non-C allele carriers.

### *IRGM* risk alleles regulate Toll-like Receptor (TLR)-induced cytokines

**Figure 3:**
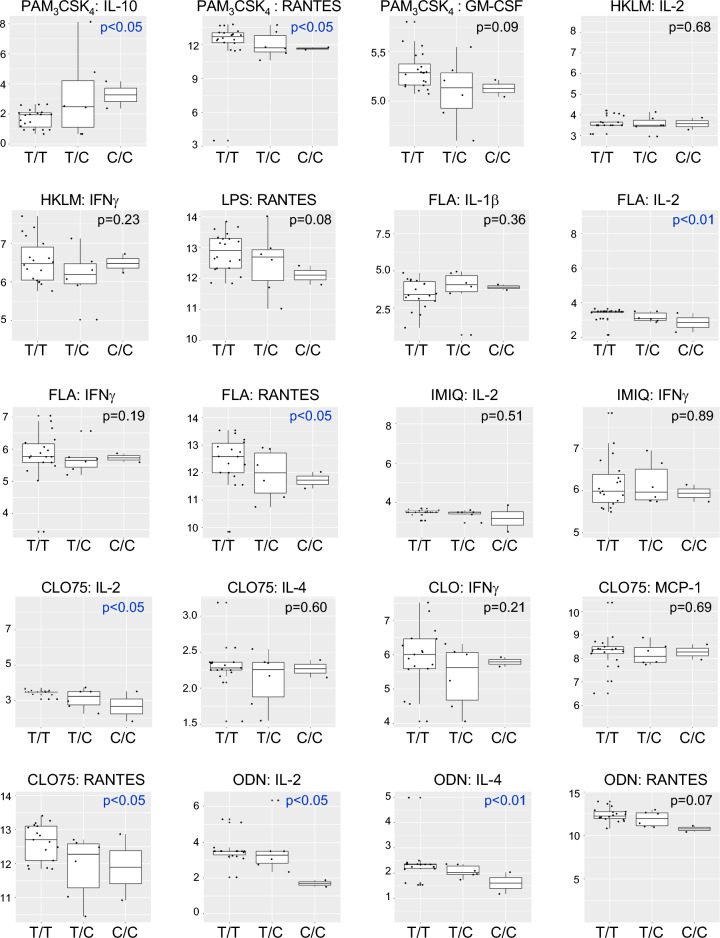
Cytokine levels in whole blood of White subjects of different rs13361189 genotypes stimulated with select TLR ligands. Whole blood from 30 White subjects of the rs13361189 genotypes shown (n = 20 [T/T]; n = 6 [T/C]; n = 2 [C/C]) was stimulated ex vivo for 3 h with the depicted Toll-like Receptor ligands, after which the cytokines indicated were quantified by multiplex assay. Box and whisker plots depict median and interquartile ranges, and y-axis units depict log-transformed pg/mL values. P values were determined by robust linear regression using a dominant genetic model (T/T vs. T/C and C/C). FLA = flagellin; GM-CSF = granulocyte macrophage-colony stimulating factor; HKLM = heat-killed *Listeria monocytogenes*; IFN = interferon; IL = interleukin; IMIQ = imiquimod; LPS = lipopolysaccharide; MCP = monocyte chemoattractant protein; ODN = oligodeoxynucleotide; RANTES = regulated on activation normal T cell expressed and secreted.

To test whether rs13361189 minor-allele carriers have altered inflammatory responsiveness, whole blood was incubated with ligands for TLR2 (Pam3CSK4, heat-killed *L. monocytogenes* [HKLM]), TLR4 (LPS), TLR5 (flagellin [FLA]), TLR7 (imiquimod [IMIQ]), TLR7/8 (CLO75), and TLR9 (oligodeoxynucleotide [ODN]) and the serum fraction assayed for cytokines. As shown in Table S4, very few genotype-dependent differences in cytokines were observed in the full study cohort. However, stratifying by race, we found that, in White subjects, a subset of TLR ligand-cytokine relationships were significantly altered in rs13361189 minor allele carriers, in almost all cases, with minor allele carriers exhibiting an attenuated response in pro-inflammatory cytokines (Table S5, Fig. 3). In response to Pam3CSK4, induction of the anti-inflammatory cytokine IL-10 was higher in minor allele carriers, whereas RANTES and GM-CSF were lower. Similar attenuated responses in minor allele carriers were observed for FLA (IL-2, RANTES), CLO (IL-2, RANTES), and ODN (IL-2, IL-4, RANTES).

**Figure 4:**
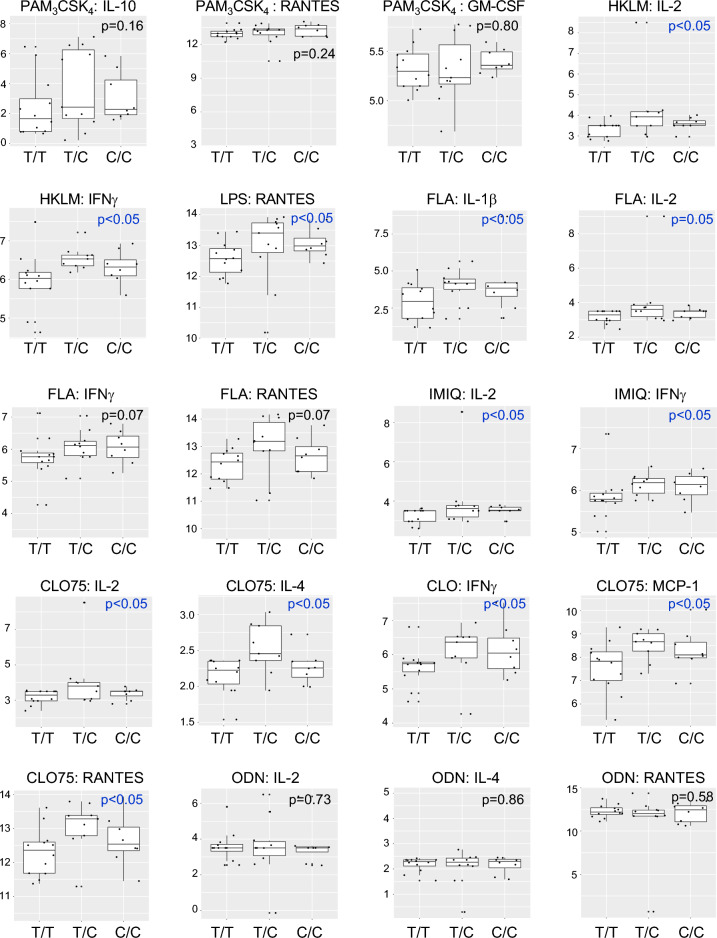
Cytokine levels in whole blood of African American subjects of different rs13361189 genotypes stimulated with select TLR ligands. Whole blood from 34 African American subjects of the rs13361189 genotypes shown (n = 12 [T/T]; n = 11 [T/C]; n = 8 [C/C]) was stimulated ex vivo for 3 h with the depicted Toll-like Receptor ligands, after which the cytokines indicated were quantified by multiplex assay. Box and whisker plots depict median and interquartile ranges, and y-axis units depict log-transformed pg/mL values. P values were determined by robust linear regression using a dominant genetic model (T/T vs. T/C and C/C). FLA = flagellin; GM-CSF = granulocyte macrophage-colony stimulating factor; HKLM = heat-killed *Listeria monocytogenes*; IFN = interferon; IL = interleukin; IMIQ = imiquimod; LPS = lipopolysaccharide; MCP = monocyte chemoattractant protein; ODN = oligodeoxynucleotide; RANTES = regulated on activation normal T cell expressed and secreted.

By contrast, among AA subjects, several TLR ligands elicited higher cytokines in minor allele carriers (Table S6, Fig. 4). These included HKLM (IL-2, IFNγ, IL-10), IMIQ (IL-2, IFNγ, IL-12), FLA (IL-1, IL-2, IFNγ, IL-10, RANTES), and CLO75 (IL-2, IFNγ, MCP-1, IL-4, RANTES). Formal interaction analysis revealed several TLR-cytokine relationships that were significantly different between White and AA subjects. Most striking in this regard was RANTES induction, which displayed opposite genotype-dependent responses between White and AA subjects across ligands for TLR2, TLR4, TLR5, and TLR7/8 (HKLM, FLA, LPS, Pam3CSK4, CLO75) (Table S7, Figs. 3, 4). A similar phenomenon was noted for IL-2 (IMIQ, FLA, Pam3CSK4, CLO75) and IFNγ (HKLM, FLA, CLO75). Taken together, minor allele status at rs13361189 is associated in White and AA subjects with opposite directional relationships to steady state and TLR-induced cytokines, with White minor allele carriers in many cases having attenuation of cytokines, whereas AA minor allele carriers exhibit increased induction of cytokines.

## Discussion

*IRGM* polymorphisms have been associated with a wide range of human disease, but the preponderance of studies reported to date have been conducted in White populations and very few have directly examined primary immune response phenotypes in subjects with varying *IRGM* genotypes. Compared to Europeans, African populations have higher *IRGM* expression and much higher *IRGM* rs13361189 CD risk allele frequency (~ 0.50 vs. 0.10 [http://useast.ensembl.org]), but lower LD of rs13361189 to the upstream CNV deletion that has been presumed to causally impact *IRGM* expression^16^, collectively suggesting that rs13361189 might associate with distinct phenotypes in White and Black subjects. Here, we report that in the overall study population C allele carriers exhibited higher serum IgM, CRP, and CD8^+^ T cell count. Race stratification revealed that the IgM and CRP changes were largely driven by White subjects, and that White minor allele carriers also displayed increased IgE and decreased frequency of circulating plasmablasts. We also found that the rs13361189 C allele associates with increases in a subset of cytokines in whole blood of AA subjects, but reduced cytokine expression in White subjects.

Intriguing race-specific differences in the amplitude of the innate immune response have recently been documented. Nearly 10% of macrophage-expressed genes exhibit ancestry-related differences in induction during infection, with subjects of African ancestry tending to have stronger inflammatory responses and more robust restriction of intracellular bacterial growth^29^. These race-specific differences are thought to arise at least in part from higher frequencies in Black than White subjects of alleles associated with increased pro-inflammatory responses^29,30^. In addition, Africans and AAs reportedly have a higher degree of genetic diversity than other racial groups^31^ and high genetic diversity of populations has been shown to be associated with more robust immune responses^32–35^. African genetic ancestry is associated with a protective effect in infections such as Dengue^36^.

Several prior examples of race-specific associations of SNPs with infectious diseases have been reported, but the molecular mechanisms have rarely been defined. For example, specific polymorphisms in *TLR1*, *TLR2*, and *TLR4* associate with tuberculosis risk in some racial groups but not others^37^, and select *TLR1* SNPs are associated with candidemia in Whites but not AAs^38^. One report also found that the rs10065172 *IRGM* CD risk allele is associated with tuberculosis in AAs but not Whites^22^. While there are fewer examples of SNPs with directionally opposite disease associations in different racial/ethnic groups, several have been reported. Thus, the A allele of TLR2 rs5743708 is associated with increased susceptibility to tuberculosis among Asian subjects, but protection among Hispanic subjects^39^. IL-6 rs1800795 is associated with allergic disease susceptibility among Asians and Caucasians in opposite directions^40^. The peroxisome proliferator-activated receptor gamma 2 Pro12Ala polymorphism has opposite associations with incident obesity in AAs and Whites^41^. In other cases, directionally opposite gene × environment interactions have been noted for polymorphisms, such as the C allele of CD14 −260, which associates with higher levels of allergic IgE in children with regular pet contact but lower levels in children regular contact with stable animals^42^.

The mechanism by which rs13361189 and other polymorphisms confer opposite phenotypic outcomes in different racial/ethnic groups is unclear, but, in principle, could be genetic and/or biochemical. Given the much lower LD of rs13361189 in African populations (r^2^ = 0.66–0.70) than European populations (r^2^ = 0.95) with a large CNV deletion upstream of *IRGM* that has been presumed to causally impact *IRGM* expression^16^, it is plausible that there is another linked, causal locus in AAs that drives the association of rs13361189 with cytokine expression. Precedence for this possibility has been shown for the *MCP-1* −2581G polymorphism, which associates with increased susceptibility to tuberculosis in Mexican and Korean subjects, but resistance to tuberculosis in Ghanaian subjects, a paradox that has been posited to arise from differences between the populations in LD of the locus to *MCP-1* −362C^43^. Although the *IRGM* upstream CNV deletion has been shown to associate with directionally opposite changes in *IRGM* expression in different cell types/lines^12^, it has been confirmed that the rs13361189 risk allele associates with reduced *IRGM* expression in African populations similarly to that in White populations^16^. Studies to date, however, have not directly addressed whether rs13361189 associates differentially with expression of the several reported isoforms of IRGM protein, much less whether it does so in a race-specific manner. Of four described splice isoforms, the IRGMd protein isoform reportedly induces mitochondrial fragmentation and cell death in a concentration-dependent manner^44^. Given reported large differences in steady state *IRGM* mRNA expression between White and AA subjects, it is plausible that allele-specific changes in protein expression of select isoforms could lead to differential effects between races on cell biology.

While our study is the first to phenotype cell-level immune function in subjects with different *IRGM* polymorphisms stratified by race, it nonetheless had several limitations. Due to limited study size, the various cytokine measurements were not adjusted for multiple testing. Although the possibility of false discovery cannot be excluded, the opposing directionality of cytokine expression between White and AA subjects across several treatment conditions was very consistent. Our study was also not adequately powered to address the possibility of sex-specific effects on cytokine expression. Cytokines were also measured in response to a single concentration of stimulus and at a single timepoint, thus not allowing for detection of dose-dependent or temporal effects.

Taken together, we report that the rs13361189 CD risk allele, which has largely been studied at the population level and predominantly in White populations, associates with intriguing changes in the immunophenotype of human subjects. Future, larger studies are warranted, as are fine mapping studies of the *IRGM* locus that might allow for better understanding of race-specific LD patterns and allelic associations to *IRGM* isoform expression. Our findings suggest that broad racial inclusion is important in functional studies of the genetic underpinnings of immunity.

## Methods

### Clinical protocol

Subjects were recruited from the NIEHS Personalized Environment and Genes Study (PEGS), formerly the Environmental Polymorphisms Registry (EPR), which is a repository of DNA and associated demographic data from a cohort of > 18,000 human subjects (~ 2/3 non-Hispanic White, ~ 1/4 non-Hispanic Black, remainder Asian, Hispanic, and Other) in North Carolina^45,46^. The study was approved by the National Institute of Environmental Health Sciences Institutional Review Board and all participants gave informed consent. All methods were performed in accordance with relevant guidelines and regulations. Exclusion criteria for the participants in the present analysis included age < 18 years, active smoking, use of anti-inflammatory (e.g., non-steroidal anti-inflammatory drugs) or immunosuppressive (e.g., corticosteroids) agents, confirmed/suspected immunodeficiency, and recent gastrointestinal or respiratory illness.

### Genotyping of *IRGM*

DNA was extracted from blood using the Autopure LS system (Qiagen) and *IRGM* SNPs were genotyped using fluorescence-based allelic TaqMan SNP Genotyping Assays (Applied Biosystems, Life Technologies, Grand Island, NY). TaqMan SNP Genotyping Assays were used, as follows: rs10065172, Assay ID C__30593568_10; rs13361189, Assay ID C__31986315_10; and rs9637876, custom assay. Polymerase chain reactions were performed using 30 ng of genomic DNA isolated from whole blood in a 10ul reaction volume using an Applied Biosystems ViiA-7 Real time PCR machine.

### Flow cytometry

In brief, anticoagulated peripheral blood was collected and immediately processed. 100uL of blood was stained with a broad immunophenotyping panel consisting of CD19 APC-Cy7, CD14 AF488, CD56 PE, CD45 PerCP-Cy5.5, CD4 PE-Cy7, CD3 V450, and CD8 BV510 antibodies (BD Biosciences), to identify CD8^+^ and CD4^+^ T lymphocytes, CD14^+^ monocytes, CD56^+^ NK cells, and CD19^+^ B lymphocytes. Neutrophils, eosinophils, and basophils were estimated based on SSC vs CD45. After 30 min on ice, the sample was treated with FACS Lyse (BD Biosciences) for 10 min at room temperature, washed, and resuspended in stain buffer. To identify B lymphocyte subsets, 900 µL of blood was lysed with ACK lysis buffer, washed twice with PBS, then stained for 30 min on ice with CD19 APC-Cy7, IgD FITC, CD27 BV786, CD38 PerCP-Cy5.5, CD24 PE-CF594, CD3 V450, and CD95 APC antibodies (BD Biosciences). Antibody details are shown in Table S8. The cells were washed and stained with Zombie Yellow Viability Dye (BioLegend) for 20 min at room temperature. Cells were washed with stain buffer and fixed. CD27^+^ switched memory, CD27- switched memory, unswitched memory, naïve, transitional, and plasmablast B lymphocyte subsets could be determined from this panel. Thirty thousand CD45^+^ cells were recorded for the immunophenotyping sample. For the B lymphocyte subset analysis, the entire sample was recorded (~ 2–3 × 10^6^ cells). Samples were acquired on a BD FACS Aria II (BD Biosciences), and analyzed using FlowJo software (Treestar, Ashland, OR). Exemplary flow cytometry gating is shown in Figs. S1 and S2.

### Ex vivo whole blood stimulation

As previously described^28^, blood was drawn from fasting subjects before 10 AM and was anticoagulated with pyrogen-free citrate (0.1 mol/L, pH 7.2), diluted 1:1 with RPMI 1640 medium, and then added to each well of a 96-well plate containing TLR ligands (1 μg/mL Pam3CSK4, 8 × 10^7^ heat-killed *Listeria monocytogenes*, 1 ng/mL *E. coli* 0111:B4 LPS, 1 μg/mL flagellin, 1 μg/mL CL075) in quadruplicate. After incubation (3 h, 37 °C, 5% CO_2_), cells were centrifuged, and supernatants were harvested and stored at − 80 °C until analysis.

### Luminex assay

Cytokines were quantified using a multiplex assay (Bio-Plex; Bio-Rad Laboratories, Hercules, CA), as previously described^28^. Cytokine values were log-transformed and batch-corrected across plates using plate controls (pooled samples present on all plates) with limma’s “removeBatchEffect” (10.1093/nar/gkv007) using plate identities as batches in R4.1.1.

### Clinical laboratory analyses

Complete blood cell count and differentiation, CRP, IgA, IgM, and IgG were run on a Roche Cobas 6000 Analyzer, and IgE by chemiluminescent immunometric assay on Siemens Immulite 2000 XPI (Siemens Healthcare Diagnostics, Flanders, NJ).

### Statistical analysis

For analysis of clinical values, variables were first tested for normality and were log-transformed if non-normal. For univariate analysis, we used Fisher’s exact test to compare categorical variables across the genotypes and ANOVA to compare continuous variables. For multivariate regression analysis, robust linear regression^47^ was used to assess the association between genotypes and cytokine levels, adjusting for age, sex, and race. The regression analysis was performed in the full study population and also separately in AA and White subjects. We also assessed genotype by race (AA vs. White) interaction effects.

### Supplementary Information

Supplementary Information.

## Data Availability

The datasets generated and/or analyzed during the current study are available from the corresponding author on reasonable request.
