# Supplementary material for: Race-specific association of an IRGM risk allele with cytokine expression in human subjects
Source: Sci Rep. 2023 Aug 9;13:12911. doi: 10.1038/s41598-023-40313-3 (PMC10412543; doi:10.1038/s41598-023-40313-3)

Race-specific association of an *IRGM* risk allele with cytokine expression in human subjects

Teminioluwa Ajayi[1], Prashant Rai[1], Min Shi[2], Kristin A. Gabor[1], Peer W. F. Karmaus[1], Julie M. Meacham[1], Kevin Katen[3], Jennifer H. Madenspacher[1], Shepherd H. Schurman[4,*], Michael B. Fessler[1]

[1]Immunity, Inflammation and Disease Laboratory, National Institute of Environmental Health Sciences, Research Triangle Park, NC 27709
[2]Biostatistics & Computational Biology Branch, National Institute of Environmental Health Sciences, Research Triangle Park, NC 27709
[3]Signal Transduction Laboratory, National Institute of Environmental Health Sciences, Research Triangle Park, NC 27709
[4]Clinical Research Branch, National Institute of Environmental Health Sciences, Research Triangle Park, NC 27709
*Current address: Clinical Research Unit, National Institute on Aging, Baltimore, MD 21225

SUPPLEMENTAL MATERIALS

Table S1. Control cytokine levels for full study population*

Analyte	Estimate	Std. Error	t value	p-value
GM-CSF	0.045	0.104	0.432	0.659
IL-1b	0.013	0.174	0.076	0.938
IL-12	-0.290	0.624	-0.465	0.644
IL-2	0.012	0.212	0.058	0.952
IFN-γ	-0.001	0.127	-0.011	0.991
IL-10	-0.106	0.250	-0.424	0.669
IL-8	0.039	0.199	0.196	0.844
IP-10	0.043	0.155	0.277	0.780
MCP-1	-0.028	0.126	-0.221	0.823
IL-4	0.014	0.102	0.136	0.890
IL-6	0.247	0.658	0.375	0.711
TNF-α	0.067	0.149	0.448	0.653
RANTES	-0.013	0.346	-0.038	0.969

*Cytokine differences were tested between rs13361189 C allele noncarriers and carriers using robust linear regression.

Table S2. Control cytokine levels in White study subjects*

Analyte	Estimate	Std. Error	t value	p-value
GM-CSF	-0.349	0.134	-2.600	0.018
TNF-a	-0.738	0.216	-3.421	0.003
IL-12	-0.944	0.516	-1.831	0.073
IL-1b	-0.588	0.229	-2.567	0.019
IL-2	-0.435	0.242	-1.798	0.097
IFNγ	-0.383	0.175	-2.194	0.043
IL-10	-0.648	0.217	-2.988	0.006
IL-8	-0.675	0.242	-2.790	0.014
IP-10	-0.238	0.151	-1.572	0.126
IL-4	-0.357	0.133	-2.684	0.013
MCP-1	-0.494	0.137	-3.604	0.002
IL-6	-2.472	0.884	-2.796	0.010
RANTES	-1.321	0.459	-2.876	0.010

*Cytokine differences were tested between rs13361189 C allele noncarriers and carriers using robust linear regression.

Table S3. Control cytokines in African American study subjects*

Analyte	Estimate	Std. Error	t value	p-value
GM-CSF	0.145	0.097	1.505	0.141
IL-1b	0.043	0.149	0.288	0.773
IL-12	0.606	0.784	0.773	0.444
IL-2	0.247	0.236	1.046	0.300
IFN-γ	0.205	0.102	2.014	0.051
IL-10	0.186	0.305	0.609	0.547
IL-8	0.070	0.220	0.316	0.751
IP-10	0.075	0.197	0.381	0.716
MCP-1	0.096	0.136	0.704	0.483
IL-4	0.189	0.085	2.232	0.031
IL-6	0.580	0.685	0.847	0.400
TNF-α	0.136	0.098	1.395	0.169
RANTES	0.539	0.242	2.223	0.033

*Cytokine differences were tested between rs13361189 C allele noncarriers and carriers using robust linear regression.

Table S4. Toll-like Receptor ligand-induced cytokine levels for full study population*

Type	Analyte	Estimate	Std. Error	t value	p-value
HKLM	GM-CSF	-0.010	0.070	-0.140	0.886
HKLM	IL-1b	-0.241	0.637	-0.378	0.708
HKLM	IL-2	0.080	0.180	0.441	0.660
HKLM	IFN-g	0.064	0.203	0.313	0.751
HKLM	IL-10	-0.002	0.246	-0.008	0.993
HKLM	IL-12	-0.070	0.137	-0.513	0.605
HKLM	IP-10	0.350	0.242	1.447	0.170
HKLM	MCP-1	-0.126	0.321	-0.392	0.702
HKLM	IL-4	0.019	0.107	0.181	0.856
HKLM	IL-6	-0.309	0.456	-0.678	0.503
HKLM	IL-8	-0.327	0.389	-0.841	0.411
HKLM	RANTES	-0.437	0.213	-2.059	0.045
HKLM	TNF-a	-0.348	0.468	-0.743	0.466
IMIQ	GM-CSF	0.194	0.087	2.234	0.028
IMIQ	IL-2	0.152	0.092	1.648	0.115
IMIQ	IFN-g	0.075	0.155	0.486	0.619
IMIQ	IL-10	0.117	0.207	0.564	0.575
IMIQ	IL-12	0.074	0.085	0.871	0.373
IMIQ	IL-1b	0.228	0.379	0.601	0.542
IMIQ	MCP-1	-0.102	0.243	-0.419	0.672
IMIQ	IL-4	-0.005	0.051	-0.105	0.916
IMIQ	IL-6	0.101	0.452	0.223	0.823
IMIQ	IP-10	0.041	0.306	0.135	0.893
IMIQ	IL-8	0.036	0.467	0.077	0.939
IMIQ	RANTES	0.125	0.235	0.532	0.592
IMIQ	TNF-a	0.087	0.262	0.334	0.735
ODN	GM-CSF	0.080	0.059	1.352	0.176
ODN	IL-12	-0.087	0.153	-0.571	0.578
ODN	IL-1b	0.332	0.503	0.660	0.512
ODN	IL-2	-0.614	0.282	-2.172	0.033
ODN	IFN-g	-0.080	0.343	-0.233	0.818
ODN	IL-10	-0.122	0.266	-0.460	0.642
ODN	IL-8	-0.156	0.363	-0.431	0.663
ODN	IP-10	0.201	0.218	0.922	0.357
ODN	MCP-1	-0.250	0.277	-0.904	0.365
ODN	IL-4	-0.089	0.112	-0.796	0.440
ODN	IL-6	0.714	0.496	1.440	0.151
ODN	TNF-a	0.187	0.647	0.289	0.766
ODN	RANTES	-0.465	0.338	-1.374	0.174
FLA	GM-CSF	0.094	0.077	1.223	0.220

FLA	IL-1b	0.283	0.507	0.559	0.581
FLA	IL-2	-0.040	0.128	-0.314	0.759
FLA	IFN-g	0.106	0.227	0.468	0.635
FLA	IL-10	0.142	0.284	0.498	0.621
FLA	IL-12	0.011	0.083	0.134	0.893
FLA	IP-10	0.350	0.162	2.158	0.035
FLA	MCP-1	0.057	0.279	0.205	0.838
FLA	IL-4	-0.039	0.078	-0.495	0.614
FLA	IL-6	-0.326	0.549	-0.595	0.562
FLA	IL-8	-0.524	0.523	-1.002	0.328
FLA	TNF-a	-0.347	0.403	-0.862	0.404
FLA	RANTES	-0.167	0.326	-0.514	0.606
LPS	GM-CSF	0.025	0.080	0.311	0.754
LPS	IL-1b	0.026	0.267	0.096	0.923
LPS	IL-12	0.491	0.467	1.052	0.289
LPS	IL-2	0.008	0.155	0.054	0.957
LPS	IFN-g	-0.058	0.114	-0.510	0.604
LPS	IL-10	0.103	0.408	0.254	0.798
LPS	IL-8	-0.324	0.322	-1.008	0.327
LPS	IP-10	-0.142	0.456	-0.311	0.753
LPS	MCP-1	-0.114	0.097	-1.185	0.232
LPS	IL-4	-0.008	0.088	-0.092	0.926
LPS	IL-6	-0.670	0.395	-1.697	0.094
LPS	TNF-a	-0.123	0.256	-0.481	0.629
LPS	RANTES	-0.011	0.299	-0.036	0.971
PAM	GM-CSF	0.025	0.079	0.315	0.749
PAM	IL-1b	0.106	0.193	0.547	0.576
PAM	IL-2	0.089	0.218	0.408	0.673
PAM	IFN-g	0.016	0.118	0.140	0.887
PAM	IL-10	0.853	0.531	1.608	0.109
PAM	IL-12	0.447	0.560	0.799	0.423
PAM	IP-10	0.180	0.177	1.019	0.305
PAM	MCP-1	0.007	0.125	0.058	0.953
PAM	IL-4	0.024	0.105	0.229	0.817
PAM	IL-6	-0.212	0.504	-0.420	0.671
PAM	IL-8	0.030	0.359	0.085	0.934
PAM	TNF-a	0.097	0.272	0.354	0.722
PAM	RANTES	-0.007	0.269	-0.027	0.978
CLO	GM-CSF	-0.003	0.054	-0.047	0.962
CLO	IL-1b	-0.176	0.504	-0.348	0.732
CLO	IL-2	-0.013	0.145	-0.090	0.932
CLO	IFN-g	0.152	0.278	0.546	0.580
CLO	IL-10	-0.087	0.245	-0.354	0.721

CLO	IL-12	0.001	0.109	0.011	0.991
CLO	IP-10	0.538	0.272	1.975	0.057
CLO	MCP-1	0.131	0.295	0.444	0.660
CLO	IL-4	-0.038	0.083	-0.458	0.640
CLO	IL-6	0.030	0.530	0.056	0.955
CLO	IL-8	-0.240	0.547	-0.438	0.657
CLO	TNF-a	-0.373	0.475	-0.785	0.448
CLO	RANTES	-0.113	0.284	-0.399	0.699

*Cytokine differences were tested between rs13361189 C allele noncarriers and carriers using robust linear regression.

Table S5. Toll-like Receptor ligand-induced cytokine levels in White study subjects*

Type	Analyte	Estimate	Std. Error	t value	p-value
HKLM	GM-CSF	0.125	0.078	1.589	0.141
HKLM	RANTES	-0.380	0.236	-1.613	0.126
HKLM	TNF-a	-0.322	0.683	-0.471	0.639
HKLM	IL-12	0.025	0.081	0.310	0.757
HKLM	IL-1b	-0.747	0.830	-0.901	0.372
HKLM	IL-2	-0.067	0.157	-0.422	0.681
HKLM	IFN-g	-0.322	0.261	-1.235	0.231
HKLM	IL-10	0.610	0.362	1.686	0.109
HKLM	IL-8	-0.168	0.440	-0.382	0.706
HKLM	IP-10	0.008	0.258	0.033	0.975
HKLM	MCP-1	0.173	0.356	0.486	0.629
HKLM	IL-6	-0.366	0.546	-0.671	0.507
HKLM	IL-4	0.077	0.119	0.652	0.519
IMIQ	GM-CSF	0.113	0.123	0.918	0.376
IMIQ	RANTES	-0.168	0.254	-0.661	0.513
IMIQ	TNF-a	-0.440	0.321	-1.370	0.180
IMIQ	IL-12	-0.013	0.054	-0.233	0.818
IMIQ	IL-1b	-0.235	0.401	-0.586	0.558
IMIQ	IL-2	-0.037	0.054	-0.692	0.514
IMIQ	IFN-g	-0.030	0.221	-0.138	0.892
IMIQ	IL-10	-0.285	0.230	-1.238	0.229
IMIQ	IL-8	-0.056	0.345	-0.163	0.871
IMIQ	MCP-1	-0.045	0.279	-0.162	0.871
IMIQ	IP-10	-0.075	0.255	-0.294	0.774
IMIQ	IL-6	-0.447	0.591	-0.756	0.454
IMIQ	IL-4	-0.044	0.046	-0.967	0.336
ODN	GM-CSF	0.065	0.083	0.782	0.449
ODN	RANTES	-0.755	0.400	-1.889	0.070
ODN	TNF-a	-0.189	0.866	-0.218	0.827
ODN	IL-10	0.195	0.312	0.625	0.552
ODN	IL-12	-0.496	0.252	-1.971	0.083
ODN	IL-1b	0.228	0.638	0.358	0.724
ODN	IL-2	-0.591	0.216	-2.731	0.016
ODN	IFN-g	-0.276	0.357	-0.775	0.444
ODN	IL-6	0.649	0.625	1.039	0.315
ODN	IL-8	-0.117	0.379	-0.309	0.759
ODN	MCP-1	-0.154	0.350	-0.441	0.661
ODN	IP-10	0.197	0.273	0.722	0.478
ODN	IL-4	-0.277	0.081	-3.405	0.003
FLA	GM-CSF	0.112	0.080	1.399	0.184

FLA	IP-10	0.076	0.152	0.502	0.620
FLA	RANTES	-0.770	0.357	-2.154	0.043
FLA	TNF-a	0.346	0.471	0.735	0.463
FLA	IL-12	-0.127	0.134	-0.945	0.351
FLA	IL-1b	0.413	0.436	0.948	0.360
FLA	IL-2	-0.340	0.099	-3.449	0.003
FLA	IFN-g	-0.230	0.173	-1.329	0.185
FLA	IL-10	0.047	0.359	0.131	0.902
FLA	IL-8	-0.020	0.588	-0.033	0.974
FLA	MCP-1	0.087	0.253	0.345	0.736
FLA	IL-6	0.010	0.822	0.012	0.990
FLA	IL-4	-0.118	0.070	-1.685	0.107
LPS	IP-10	0.276	0.466	0.593	0.554
LPS	GM-CSF	-0.028	0.083	-0.339	0.736
LPS	TNF-a	0.105	0.257	0.408	0.683
LPS	IL-12	-0.179	0.424	-0.422	0.679
LPS	IL-1b	0.147	0.277	0.530	0.610
LPS	IL-2	-0.031	0.128	-0.243	0.804
LPS	IFN-g	-0.002	0.142	-0.012	0.990
LPS	IL-10	0.068	0.375	0.182	0.861
LPS	IL-8	-0.118	0.386	-0.305	0.761
LPS	IL-4	-0.028	0.108	-0.254	0.801
LPS	MCP-1	0.085	0.108	0.794	0.446
LPS	IL-6	0.352	0.458	0.770	0.447
LPS	RANTES	-0.537	0.295	-1.819	0.084
PAM	IP-10	-0.031	0.182	-0.173	0.863
PAM	GM-CSF	-0.146	0.080	-1.811	0.089
PAM	RANTES	-0.851	0.407	-2.093	0.048
PAM	TNF-a	0.249	0.308	0.808	0.424
PAM	IL-12	-0.399	0.577	-0.692	0.491
PAM	IL-1b	0.108	0.205	0.527	0.615
PAM	IL-2	-0.423	0.246	-1.718	0.105
PAM	IFN-g	-0.126	0.151	-0.839	0.412
PAM	IL-10	1.094	0.399	2.744	0.021
PAM	IL-8	0.252	0.425	0.593	0.559
PAM	MCP-1	-0.095	0.152	-0.622	0.548
PAM	IL-4	-0.188	0.122	-1.548	0.131
PAM	IL-6	0.928	0.668	1.389	0.174
CLO	GM-CSF	0.053	0.078	0.677	0.511
CLO	IP-10	-0.054	0.342	-0.159	0.874
CLO	RANTES	-0.772	0.292	-2.640	0.015
CLO	TNF-a	-0.110	0.641	-0.172	0.864
CLO	IL-12	-0.117	0.108	-1.088	0.299

CLO	IL-1b	0.006	0.437	0.013	0.990
CLO	IL-2	-0.310	0.107	-2.910	0.021
CLO	IFN-g	-0.486	0.382	-1.272	0.212
CLO	IL-10	-0.458	0.257	-1.782	0.091
CLO	IL-8	-0.287	0.580	-0.494	0.628
CLO	MCP-1	-0.085	0.213	-0.401	0.689
CLO	IL-6	-0.275	0.588	-0.467	0.641
CLO	IL-4	-0.039	0.071	-0.549	0.597

*Cytokine differences were tested between rs13361189 C allele noncarriers and carriers using robust linear regression.

Table S6. Toll-like Receptor ligand-induced cytokines in African American study subjects*

Type	Analyte	Estimate	Std. Error	t value	p-value
HKLM	GM-CSF	0.065	0.053	1.226	0.238
HKLM	IL-1b	-0.386	0.609	-0.635	0.529
HKLM	IL-2	0.409	0.178	2.297	0.029
HKLM	IFN-g	0.453	0.169	2.679	0.014
HKLM	IL-10	0.309	0.164	1.879	0.073
HKLM	IL-12	0.152	0.186	0.819	0.421
HKLM	IP-10	0.094	0.285	0.330	0.746
HKLM	MCP-1	-0.265	0.390	-0.680	0.500
HKLM	IL-4	0.191	0.120	1.592	0.128
HKLM	IL-6	-0.271	0.484	-0.561	0.579
HKLM	IL-8	-0.352	0.431	-0.816	0.422
HKLM	RANTES	0.420	0.238	1.765	0.088
HKLM	TNF-a	-0.496	0.478	-1.036	0.305
IMIQ	GM-CSF	0.129	0.088	1.471	0.155
IMIQ	IL-2	0.285	0.120	2.370	0.026
IMIQ	IFN-g	0.287	0.122	2.354	0.030
IMIQ	IL-10	0.254	0.183	1.391	0.179
IMIQ	IL-12	0.170	0.092	1.843	0.076
IMIQ	IL-1b	-0.242	0.452	-0.536	0.599
IMIQ	MCP-1	0.065	0.250	0.260	0.798
IMIQ	IL-4	0.092	0.071	1.297	0.220
IMIQ	IL-6	-0.405	0.472	-0.859	0.399
IMIQ	IP-10	0.141	0.341	0.414	0.682
IMIQ	IL-8	-0.466	0.575	-0.811	0.420
IMIQ	RANTES	0.080	0.316	0.254	0.801
IMIQ	TNF-a	0.091	0.222	0.411	0.691
ODN	GM-CSF	0.077	0.061	1.264	0.217
ODN	IL-12	-0.209	0.139	-1.510	0.141
ODN	IL-1b	0.417	0.466	0.894	0.379
ODN	IL-2	-0.116	0.329	-0.352	0.729
ODN	IFN-g	0.248	0.357	0.695	0.492
ODN	IL-10	-0.171	0.243	-0.703	0.491
ODN	IL-8	0.178	0.392	0.455	0.654
ODN	IP-10	-0.138	0.230	-0.597	0.564
ODN	MCP-1	0.285	0.326	0.874	0.394
ODN	IL-4	0.018	0.103	0.179	0.858
ODN	IL-6	0.832	0.442	1.881	0.072
ODN	TNF-a	0.524	0.464	1.131	0.272
ODN	RANTES	-0.189	0.340	-0.556	0.583

FLA	GM-CSF	-0.156	0.090	-1.747	0.096
FLA	IL-1b	1.063	0.499	2.130	0.042
FLA	IL-2	0.282	0.140	2.009	0.053
FLA	IFN-g	0.346	0.182	1.901	0.072
FLA	IL-10	0.428	0.221	1.937	0.065
FLA	IL-12	0.053	0.093	0.574	0.567
FLA	IP-10	-0.006	0.264	-0.021	0.984
FLA	MCP-1	0.387	0.364	1.062	0.302
FLA	IL-4	0.094	0.092	1.020	0.327
FLA	IL-6	-0.186	0.567	-0.328	0.745
FLA	IL-8	-0.325	0.657	-0.494	0.624
FLA	TNF-a	-0.165	0.447	-0.370	0.713
FLA	RANTES	0.587	0.317	1.850	0.073
LPS	GM-CSF	0.081	0.081	0.994	0.326
LPS	IL-1b	0.259	0.333	0.778	0.442
LPS	IL-12	0.536	0.600	0.893	0.381
LPS	IL-2	0.117	0.177	0.660	0.510
LPS	IFN-g	0.100	0.111	0.905	0.370
LPS	IL-10	0.545	0.503	1.083	0.291
LPS	IL-8	-0.210	0.398	-0.527	0.599
LPS	IP-10	-0.040	0.533	-0.075	0.941
LPS	MCP-1	0.022	0.129	0.172	0.865
LPS	IL-4	0.115	0.080	1.443	0.158
LPS	IL-6	0.036	0.519	0.069	0.945
LPS	TNF-a	-0.147	0.345	-0.425	0.674
LPS	RANTES	0.605	0.243	2.491	0.018
PAM	GM-CSF	0.025	0.101	0.248	0.805
PAM	IL-1b	0.042	0.194	0.216	0.831
PAM	IL-2	0.102	0.203	0.503	0.614
PAM	IFN-g	0.153	0.098	1.557	0.128
PAM	IL-10	1.137	0.786	1.447	0.161
PAM	IL-12	0.155	0.600	0.259	0.797
PAM	IP-10	-0.281	0.267	-1.049	0.311
PAM	MCP-1	-0.028	0.120	-0.231	0.818
PAM	IL-4	0.111	0.105	1.054	0.299
PAM	IL-6	-0.058	0.489	-0.118	0.907
PAM	IL-8	-0.113	0.453	-0.250	0.804
PAM	TNF-a	-0.150	0.294	-0.509	0.615
PAM	RANTES	0.257	0.213	1.204	0.237
CLO	GM-CSF	0.016	0.035	0.441	0.668
CLO	IL-1b	0.929	0.685	1.357	0.186
CLO	IL-2	0.337	0.160	2.099	0.044
CLO	IFN-g	0.564	0.245	2.306	0.030

CLO	IL-10	0.338	0.295	1.148	0.261
CLO	IL-12	0.065	0.110	0.589	0.558
CLO	IP-10	0.548	0.366	1.498	0.156
CLO	MCP-1	0.856	0.388	2.205	0.038
CLO	IL-4	0.225	0.108	2.090	0.045
CLO	IL-6	1.043	0.696	1.498	0.149
CLO	IL-8	0.813	0.549	1.481	0.150
CLO	TNF-a	0.524	0.590	0.889	0.383
CLO	RANTES	0.633	0.304	2.083	0.047

*Cytokine differences were tested between rs13361189 C allele noncarriers and carriers using robust linear regression.

Table S7. Race interactions in Toll-like Receptor ligand-induced cytokine levels*

Type	Analyte	Estimate	Std. Error	t value	p-value
HKLM	GM-CSF	0.080	0.108	0.742	0.471
HKLM	IL-1b	-0.535	1.114	-0.480	0.629
HKLM	IL-2	-0.379	0.221	-1.716	0.089
HKLM	IFN-g	-0.604	0.301	-2.007	0.054
HKLM	IL-10	0.241	0.389	0.619	0.544
HKLM	IL-12	-0.153	0.220	-0.698	0.483
HKLM	IP-10	-0.087	0.405	-0.214	0.832
HKLM	MCP-1	0.439	0.540	0.812	0.418
HKLM	IL-4	-0.092	0.161	-0.570	0.570
HKLM	IL-6	0.013	0.724	0.018	0.985
HKLM	IL-8	0.228	0.606	0.376	0.705
HKLM	RANTES	-0.759	0.370	-2.051	0.045
HKLM	TNF-a	0.071	0.785	0.090	0.927
IMIQ	GM-CSF	0.043	0.148	0.291	0.774
IMIQ	IL-2	-0.261	0.150	-1.743	0.087
IMIQ	IFN-g	-0.344	0.236	-1.456	0.155
IMIQ	IL-10	-0.522	0.307	-1.704	0.096
IMIQ	IL-12	-0.171	0.105	-1.624	0.113
IMIQ	IL-1b	0.052	0.609	0.085	0.932
IMIQ	MCP-1	0.046	0.371	0.124	0.901
IMIQ	IL-4	-0.113	0.088	-1.283	0.202
IMIQ	IL-6	0.518	0.721	0.718	0.474
IMIQ	IP-10	0.159	0.540	0.294	0.772
IMIQ	IL-8	0.615	0.690	0.892	0.371
IMIQ	RANTES	-0.245	0.392	-0.625	0.531
IMIQ	TNF-a	-0.516	0.412	-1.252	0.215
ODN	GM-CSF	0.024	0.101	0.239	0.812
ODN	IL-12	-0.315	0.278	-1.131	0.281
ODN	IL-1b	-0.097	0.793	-0.122	0.904
ODN	IL-2	-0.553	0.348	-1.588	0.120
ODN	IFN-g	-0.418	0.525	-0.795	0.428
ODN	IL-10	0.158	0.445	0.356	0.725
ODN	IL-8	-0.200	0.560	-0.358	0.721
ODN	IP-10	0.422	0.367	1.151	0.269
ODN	MCP-1	-0.618	0.449	-1.377	0.172
ODN	IL-4	-0.293	0.139	-2.114	0.039
ODN	IL-6	-0.035	0.803	-0.043	0.966
ODN	TNF-a	-0.799	0.961	-0.831	0.410
ODN	RANTES	-0.678	0.535	-1.267	0.207

FLA	GM-CSF	0.268	0.128	2.103	0.043
FLA	IL-1b	-0.752	0.715	-1.051	0.298
FLA	IL-2	-0.488	0.197	-2.483	0.016
FLA	IFN-g	-0.627	0.273	-2.294	0.026
FLA	IL-10	-0.360	0.406	-0.888	0.419
FLA	IL-12	-0.143	0.170	-0.842	0.405
FLA	IP-10	0.118	0.273	0.433	0.672
FLA	MCP-1	-0.164	0.453	-0.361	0.719
FLA	IL-4	-0.212	0.121	-1.755	0.085
FLA	IL-6	0.053	0.913	0.058	0.954
FLA	IL-8	0.185	0.871	0.213	0.835
FLA	TNF-a	0.347	0.698	0.497	0.616
FLA	RANTES	-1.404	0.503	-2.794	0.007
LPS	GM-CSF	-0.122	0.121	-1.004	0.316
LPS	IL-1b	-0.089	0.459	-0.195	0.849
LPS	IL-12	-0.514	0.755	-0.681	0.497
LPS	IL-2	-0.150	0.233	-0.642	0.511
LPS	IFN-g	-0.093	0.182	-0.511	0.610
LPS	IL-10	-0.411	0.643	-0.639	0.535
LPS	IL-8	-0.039	0.504	-0.078	0.938
LPS	IP-10	0.287	0.781	0.368	0.713
LPS	MCP-1	0.073	0.164	0.443	0.666
LPS	IL-4	-0.108	0.141	-0.763	0.443
LPS	IL-6	0.320	0.707	0.453	0.650
LPS	TNF-a	0.157	0.434	0.363	0.716
LPS	RANTES	-1.200	0.388	-3.093	0.003
PAM	GM-CSF	-0.192	0.127	-1.506	0.137
PAM	IL-1b	0.159	0.304	0.524	0.606
PAM	IL-2	-0.564	0.329	-1.715	0.096
PAM	IFN-g	-0.292	0.185	-1.580	0.118
PAM	IL-10	0.443	1.001	0.443	0.664
PAM	IL-12	-0.498	0.824	-0.604	0.545
PAM	IP-10	0.085	0.308	0.275	0.786
PAM	MCP-1	-0.055	0.195	-0.283	0.783
PAM	IL-4	-0.298	0.160	-1.856	0.066
PAM	IL-6	1.313	0.801	1.639	0.106
PAM	IL-8	0.466	0.627	0.744	0.458
PAM	TNF-a	0.357	0.463	0.770	0.439
PAM	RANTES	-1.148	0.384	-2.990	0.004
CLO	GM-CSF	0.049	0.094	0.518	0.605
CLO	IL-1b	-1.207	0.879	-1.373	0.175
CLO	IL-2	-0.568	0.235	-2.421	0.022
CLO	IFN-g	-1.155	0.418	-2.765	0.008

CLO	IL-10	-0.689	0.374	-1.842	0.077
CLO	IL-12	-0.186	0.166	-1.124	0.273
CLO	IP-10	-0.685	0.513	-1.337	0.192
CLO	MCP-1	-0.872	0.415	-2.102	0.040
CLO	IL-4	-0.252	0.153	-1.648	0.109
CLO	IL-6	-1.246	0.909	-1.370	0.174
CLO	IL-8	-1.075	0.839	-1.281	0.206
CLO	TNF-a	-1.113	0.932	-1.194	0.237
CLO	RANTES	-1.521	0.411	-3.698	0.001

*Cytokine levels in White rs13361889 C allele carriers minus non-C allele carriers vs. cytokine levels in African American C allele carriers minus non-C allele carriers.

Table S8. Flow cytometry antibodies and reagents

B Lymphocyte Panel				
**Target**	**Fluorochrome**	**Clone**	**Vendor**	**Catalog #**
CD19	APC-Cy7	SJ25C1	BD Bioscience	557791
IgD	FITC	IA6-2	BD Bioscience	555778
CD27	BV786	L128	BD Bioscience	563327
CD38	PerCP Cy5.5	HIT2	BD Bioscience	551400
CD24	PE-CF594	ML5	BD Bioscience	562405
CD3	V450	UCHT1	BD Bioscience	560365
CD95	APC	DX2	BD Bioscience	558814
Live/Dead	Zombie Yellow		Biolegend	423103

Leukocyte Phenotype Panel				
**Target**	**Fluorochrome**	**Clone**	**Vendor**	**Catalog #**
CD19	APC-Cy7	SJ25C1	BD Bioscience	557791
CD14	AlexaFluor 488	M5E2	BD Bioscience	557700
CD56	PE	B159	BD Bioscience	555516
CD45	PerCP Cy5.5	HI30	BD Bioscience	564105
CD4	PE-Cy7	SK3	BD Bioscience	557852
CD3	V450	UCHT1	BD Bioscience	560365
CD8	BV510	SK1	BD Bioscience	563919

FACS Lysing Solution		BD Bioscience	349202

Figure S1. Exemplary flow cytometry gating for leukocyte staining panel.

Figure S2. Exemplary flow cytometry gating for B cell staining panel.

Leukocyte Staining Panel

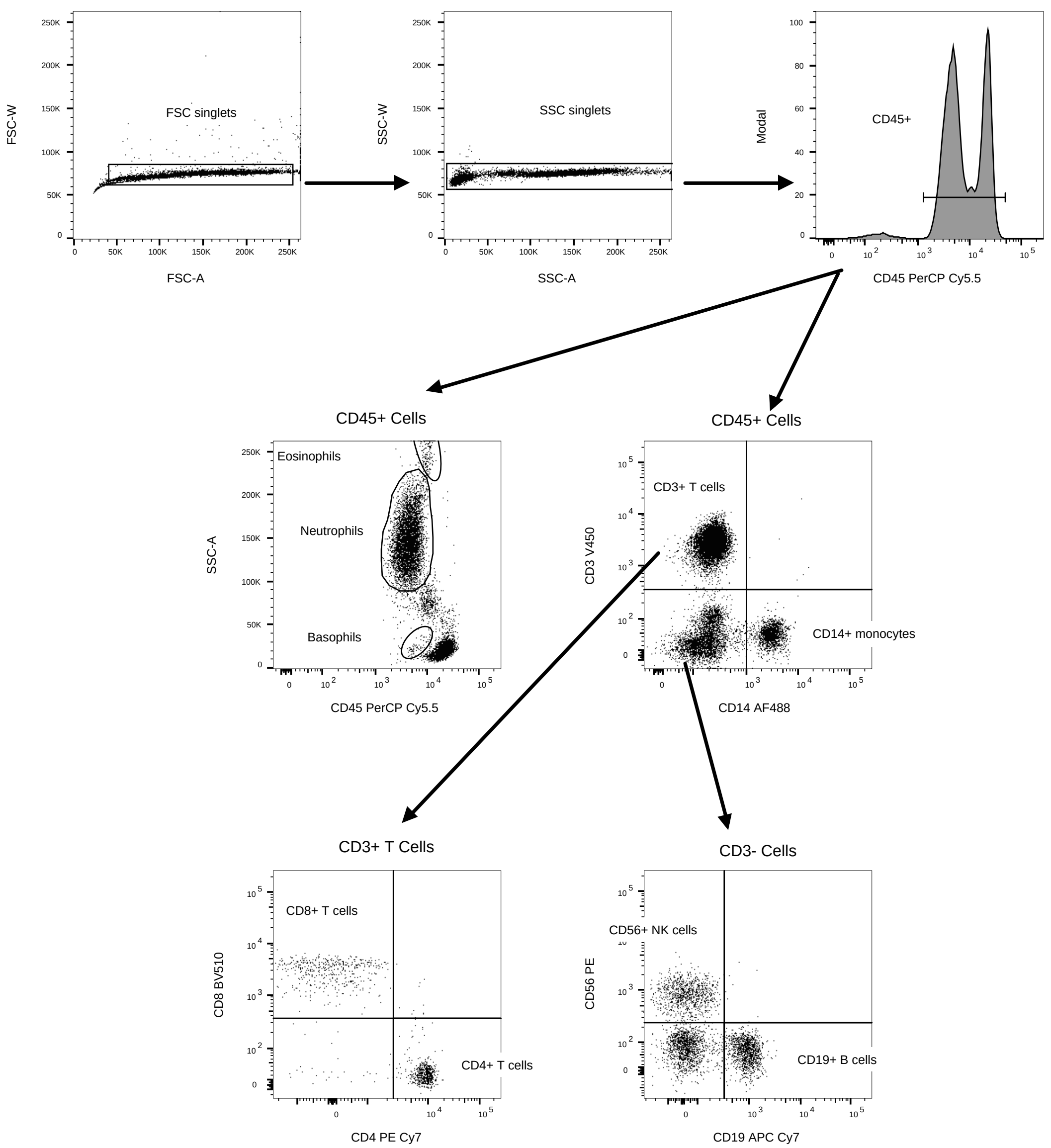

B Lymphocyte Staining Panel

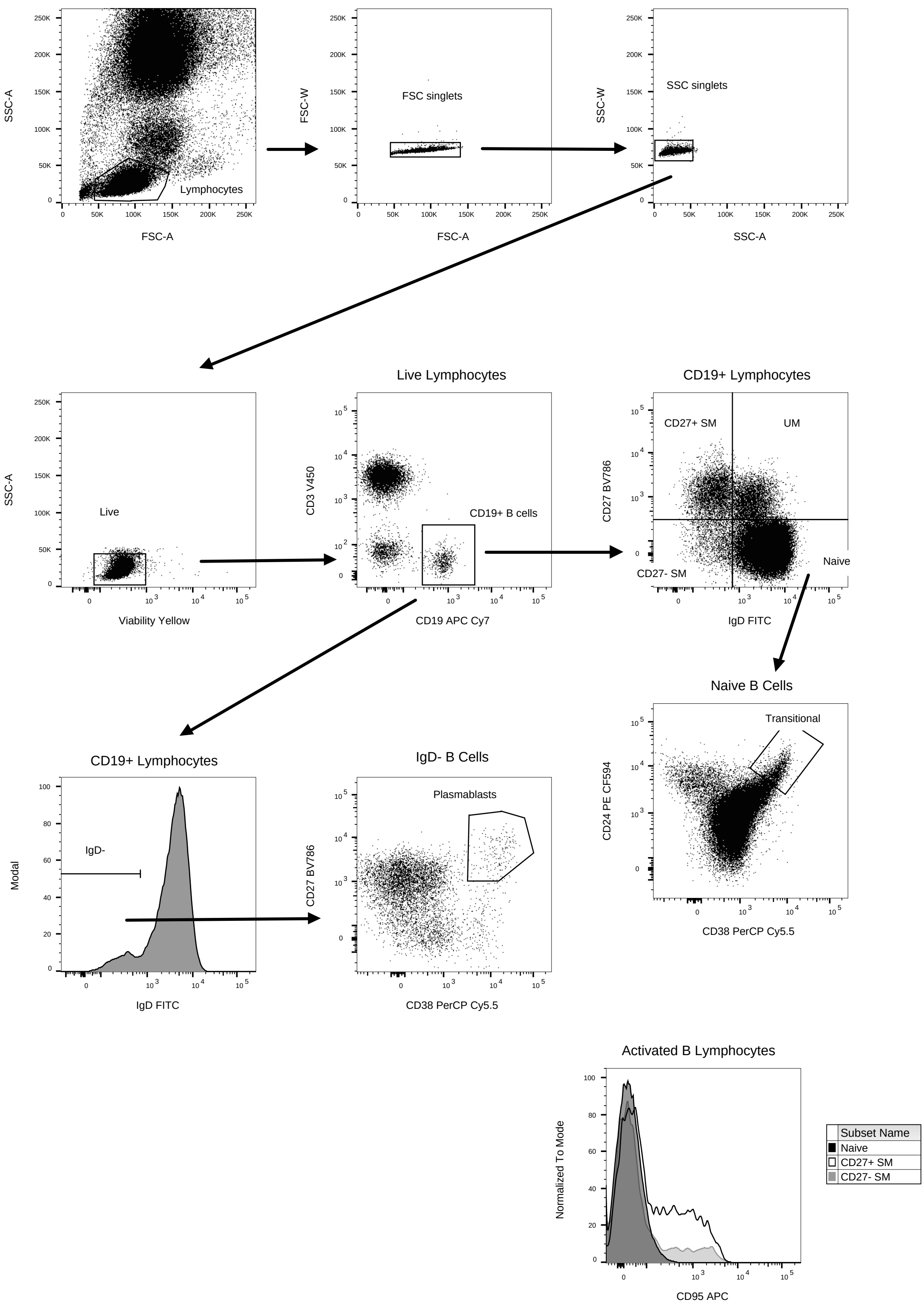

Supplement: Supplementary file 1 — Supplementary Information. [file 41598_2023_40313_MOESM1_ESM.pdf]
